# 14-Hy­droxy-8,14-secogammacera-7-ene-3,21-dione from the bark of *Lansium domesticum* Corr.

**DOI:** 10.1107/S1600536810021136

**Published:** 2010-06-09

**Authors:** Unang Supratman, Tri Mayanti, Khalijah Awang, Mat Ropi Mukhtar, Seik Weng Ng

**Affiliations:** a Department of Chemistry, Faculty of Mathematics and Natural Sciences, Padjadjaran University, Jatinangor 45363, Indonesia; b Department of Chemistry, University of Malaya, 50603 Kuala Lumpur, Malaysia

## Abstract

In the title compound (kokosanolide B), C_30_H_48_O_3_, the hexa­hydro- and octa­hydro­naphthalen-2-one ring systems are connected through an ethyl­ene fragment, with a C—CH_2_—CH_2_—C torsion angle of 176.2 (2)°. The cyclo­hexene ring adopts a half-chair conformation, while the other six-membered rings adopt distorted chair conformations. In the crystal, adjacent mol­ecules are linked into a zigzag chain along the *b* axis by O—H⋯O hydrogen bonds involving the hy­droxy and carbonyl groups.

## Related literature

For a related compound from the same species, see: Tjokronegero *et al.* (2009 ▶). For kokosanolide A, see: Mayanti *et al.* (2009 ▶).
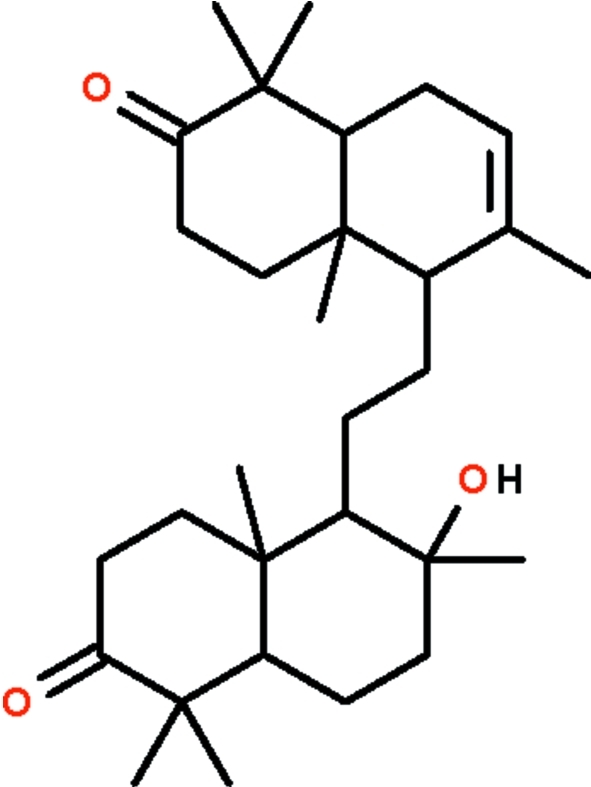


## Experimental

### 

#### Crystal data

C_30_H_48_O_3_
*M* *_r_* = 456.68
Orthorhombic, 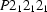

*a* = 11.8841 (11) Å
*b* = 14.8301 (13) Å
*c* = 15.2755 (13) Å
*V* = 2692.2 (4) Å^3^
*Z* = 4
Mo *K*α radiation
μ = 0.07 mm^−1^
*T* = 100 K
0.20 × 0.10 × 0.05 mm

#### Data collection

Bruker SMART APEXII diffractometer
26171 measured reflections
3469 independent reflections
3033 reflections with *I* > 2σ(*I*)
*R*_int_ = 0.067

#### Refinement

*R*[*F* ^2^ > 2σ(*F* ^2^)] = 0.039
*wR*(*F* ^2^) = 0.098
*S* = 1.01
3469 reflections
310 parameters
1 restraint
H atoms treated by a mixture of independent and constrained refinement
Δρ_max_ = 0.25 e Å^−3^
Δρ_min_ = −0.20 e Å^−3^

### 

Data collection: *APEX2* (Bruker, 2009 ▶); cell refinement: *SAINT* (Bruker, 2009 ▶); data reduction: *SAINT*; program(s) used to solve structure: *SHELXS97* (Sheldrick, 2008 ▶); program(s) used to refine structure: *SHELXL97* (Sheldrick, 2008 ▶); molecular graphics: *X-SEED* (Barbour, 2001 ▶); software used to prepare material for publication: *publCIF* (Westrip, 2010 ▶).

## Supplementary Material

Crystal structure: contains datablocks global, I. DOI: 10.1107/S1600536810021136/ci5095sup1.cif

Structure factors: contains datablocks I. DOI: 10.1107/S1600536810021136/ci5095Isup2.hkl

Additional supplementary materials:  crystallographic information; 3D view; checkCIF report

## Figures and Tables

**Table 1:** Hydrogen-bond geometry (Å, °)

*D*—H⋯*A*	*D*—H	H⋯*A*	*D*⋯*A*	*D*—H⋯*A*
O2—H2⋯O3^i^	0.84 (1)	2.15 (1)	2.974 (2)	167 (3)

Symmetry code: (i) 
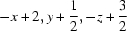
.

**Fig. 1.**
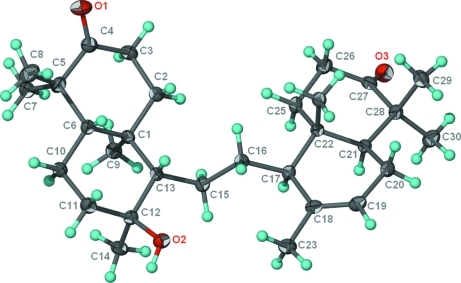
Displacement ellipsoid plot (Barbour, 2001) of C30H48O3 at the 70% probability level. H atoms are drawn as spheres of arbitrary radius.

## supplementary crystallographic information

### Comment

A previous study on the bark of *Lansium domesticum *Corr (Meliaceae) yielded a co-crystal, 8,14-secogammacera-7,14 (27)-diene–3,21-dione-8,14-secogammacera-7,14-diene-3,21-dione (1.5/0.5). The major component has an excocyclic double bond and an endocyclic double bond (Tjokronegero *et al.*, 2009). In the present compound (Scheme I), a molecule of water has been added across the endocyclic double bond to furnish the corresponding alcohol (Fig. 1).

The hexahydro-naphthalen-2-one and octahydro-naphthalen-2-one ring systems are connected through an ethylene fragment, with a C–CH_2_–CH_2_–C torsion angle of 176.2 (2)°. The hydroxy unit of one fused-ring forms a hydrogen bond to the ketonic unit of the other fused-ring of an adjacent molecule to generate a zigzag chain.

### Experimental

*Lansium domesticum * Corr. (Meliaceae) was collected in Cililin, Bandung, Indonesia, in 2006. The plant was identified by the staff at Department of Biology, Padjadjaran University. The dried and milled bark of *L. domesticum* (3 kg) was extracted exhaustively by methanol at room temperature. The methanol extract (250 g) was partitioned between *n*-hexane and ethyl acetate to give an *n*-hexane soluble fraction (70 g) and an ethyl acetate soluble fraction (40 g). The ethyl acetate fraction was subjected to vacuum column chromatography on silica gel 60 by using a step gradient of *n*-hexane/ethyl acetate/methanol. The fraction eluted by *n*-hexane/ethyl acetate (80:20) was further separated by column chromatography on silica gel *n*-hexane/ethyl acetate (95:5) and *n*-hexane/acetone (90:10). Single crystals were obtained by slow evaporation of the solvent.

### Refinement

Carbon-bound H-atoms were placed in calculated positions [C—H = 0.95–1.00 Å] and were included in the refinement in the riding-model approximation, with *U_iso_*(H) = 1.2–1.5*U*(C). The hydroxy H-atom was located in a difference Fourier map and was refined with a distance restraint of O–H = 0.84 (1) Å; its U_iso_ parameter was freely refined. 2721 Friedel pairs were merged

### Crystal data

**Table:**

C_30_H_48_O_3_	*F*(000) = 1008
*M**_r_* = 456.68	*D*_x_ = 1.127 Mg m^−^^3^
Orthorhombic, *P*2_1_2_1_2_1_	Mo *K*α radiation, λ = 0.71073 Å
Hall symbol: P 2ac 2ab	Cell parameters from 3379 reflections
*a* = 11.8841 (11) Å	θ = 2.6–20.6°
*b* = 14.8301 (13) Å	µ = 0.07 mm^−^^1^
*c* = 15.2755 (13) Å	*T* = 100 K
*V* = 2692.2 (4) Å^3^	Plate, colourless
*Z* = 4	0.20 × 0.10 × 0.05 mm

### Data collection

**Table:**

Bruker SMART APEXII diffractometer	3033 reflections with *I* > 2σ(*I*)
Radiation source: fine-focus sealed tube	*R*_int_ = 0.067
graphite	θ_max_ = 27.5°, θ_min_ = 1.9°
ω scans	*h* = −15→15
26171 measured reflections	*k* = −17→19
3469 independent reflections	*l* = −19→19

### Refinement

**Table:**

Refinement on *F*^2^	Primary atom site location: structure-invariant direct methods
Least-squares matrix: full	Secondary atom site location: difference Fourier map
*R*[*F*^2^ > 2σ(*F*^2^)] = 0.039	Hydrogen site location: inferred from neighbouring sites
*wR*(*F*^2^) = 0.098	H atoms treated by a mixture of independent and constrained refinement
*S* = 1.01	*w* = 1/[σ^2^(*F*_o_^2^) + (0.0534*P*)^2^ + 0.4471*P*] where *P* = (*F*_o_^2^ + 2*F*_c_^2^)/3
3469 reflections	(Δ/σ)_max_ = 0.001
310 parameters	Δρ_max_ = 0.25 e Å^−^^3^
1 restraint	Δρ_min_ = −0.20 e Å^−^^3^

### Fractional atomic coordinates and isotropic or equivalent isotropic displacement parameters (Å^2^)

**Table:**

	*x*	*y*	*z*	*U*_iso_*/*U*_eq_	
O1	0.85660 (14)	0.46079 (11)	1.07799 (11)	0.0313 (4)	
O2	0.92757 (12)	0.95328 (10)	1.00483 (10)	0.0193 (3)	
H2	0.956 (2)	0.9990 (13)	1.0283 (17)	0.044 (9)*	
O3	0.97661 (13)	0.62965 (10)	0.44013 (10)	0.0230 (3)	
C1	1.00012 (16)	0.70418 (13)	0.99742 (13)	0.0139 (4)	
C2	0.94385 (17)	0.63545 (13)	0.93607 (13)	0.0171 (4)	
H2A	0.8627	0.6497	0.9315	0.021*	
H2B	0.9770	0.6414	0.8769	0.021*	
C3	0.95721 (19)	0.53755 (14)	0.96705 (14)	0.0205 (5)	
H3A	0.9141	0.4974	0.9276	0.025*	
H3B	1.0375	0.5202	0.9632	0.025*	
C4	0.91696 (17)	0.52388 (14)	1.05964 (15)	0.0190 (4)	
C5	0.95844 (18)	0.59017 (14)	1.12907 (14)	0.0188 (4)	
C6	0.95358 (17)	0.68799 (13)	1.09158 (13)	0.0155 (4)	
H6	0.8714	0.7017	1.0870	0.019*	
C7	1.07759 (19)	0.55946 (15)	1.15649 (15)	0.0234 (5)	
H7A	1.0735	0.4990	1.1822	0.035*	
H7B	1.1081	0.6017	1.1997	0.035*	
H7C	1.1267	0.5582	1.1049	0.035*	
C8	0.8817 (2)	0.58287 (17)	1.20997 (15)	0.0296 (5)	
H8A	0.8724	0.5193	1.2259	0.044*	
H8B	0.8080	0.6091	1.1965	0.044*	
H8C	0.9158	0.6156	1.2590	0.044*	
C9	1.12928 (17)	0.69324 (14)	0.98960 (14)	0.0185 (4)	
H9A	1.1478	0.6293	0.9820	0.028*	
H9B	1.1653	0.7160	1.0429	0.028*	
H9C	1.1565	0.7275	0.9390	0.028*	
C10	0.99922 (19)	0.75933 (14)	1.15478 (13)	0.0199 (4)	
H10A	0.9721	0.7465	1.2148	0.024*	
H10B	1.0825	0.7571	1.1553	0.024*	
C11	0.96049 (19)	0.85294 (14)	1.12704 (14)	0.0204 (4)	
H11A	0.9925	0.8980	1.1679	0.025*	
H11B	0.8775	0.8561	1.1318	0.025*	
C12	0.99487 (18)	0.87777 (13)	1.03345 (13)	0.0162 (4)	
C13	0.96039 (16)	0.80090 (13)	0.97016 (12)	0.0133 (4)	
H13	0.8765	0.7983	0.9749	0.016*	
C14	1.11914 (18)	0.90456 (14)	1.03022 (15)	0.0212 (5)	
H14A	1.1428	0.9110	0.9691	0.032*	
H14B	1.1645	0.8578	1.0587	0.032*	
H14C	1.1296	0.9620	1.0608	0.032*	
C15	0.98231 (16)	0.82616 (14)	0.87297 (13)	0.0160 (4)	
H15A	1.0086	0.8894	0.8698	0.019*	
H15B	1.0428	0.7872	0.8495	0.019*	
C16	0.87697 (17)	0.81557 (14)	0.81600 (13)	0.0159 (4)	
H16A	0.8152	0.8511	0.8425	0.019*	
H16B	0.8539	0.7514	0.8166	0.019*	
C17	0.89186 (16)	0.84596 (14)	0.71915 (12)	0.0152 (4)	
H17	0.9747	0.8520	0.7092	0.018*	
C18	0.84100 (17)	0.93890 (14)	0.70231 (13)	0.0168 (4)	
C19	0.78520 (18)	0.95775 (14)	0.62919 (14)	0.0184 (4)	
H19	0.7511	1.0155	0.6245	0.022*	
C20	0.77245 (18)	0.89399 (14)	0.55361 (13)	0.0178 (4)	
H20A	0.7846	0.9270	0.4981	0.021*	
H20B	0.6950	0.8694	0.5530	0.021*	
C21	0.85675 (16)	0.81635 (13)	0.56021 (13)	0.0138 (4)	
H21	0.9325	0.8456	0.5579	0.017*	
C22	0.84927 (16)	0.77380 (13)	0.65296 (13)	0.0133 (4)	
C23	0.8624 (2)	1.01122 (15)	0.76957 (15)	0.0237 (5)	
H23A	0.8226	1.0664	0.7527	0.036*	
H23B	0.9434	1.0234	0.7729	0.036*	
H23C	0.8353	0.9909	0.8268	0.036*	
C24	0.72842 (17)	0.74445 (14)	0.67586 (13)	0.0165 (4)	
H24A	0.7286	0.7126	0.7321	0.025*	
H24B	0.6998	0.7043	0.6300	0.025*	
H24C	0.6801	0.7978	0.6801	0.025*	
C25	0.92925 (17)	0.69270 (14)	0.65756 (13)	0.0171 (4)	
H25A	1.0077	0.7143	0.6521	0.021*	
H25B	0.9214	0.6635	0.7155	0.021*	
C26	0.90659 (19)	0.62292 (14)	0.58610 (13)	0.0194 (4)	
H26A	0.9602	0.5722	0.5922	0.023*	
H26B	0.8295	0.5987	0.5929	0.023*	
C27	0.91895 (17)	0.66457 (14)	0.49686 (13)	0.0166 (4)	
C28	0.85347 (18)	0.75171 (14)	0.47911 (12)	0.0163 (4)	
C29	0.73416 (19)	0.72086 (16)	0.45128 (14)	0.0226 (5)	
H29A	0.7391	0.6859	0.3970	0.034*	
H29B	0.6865	0.7739	0.4418	0.034*	
H29C	0.7014	0.6833	0.4975	0.034*	
C30	0.9062 (2)	0.80059 (15)	0.40058 (14)	0.0240 (5)	
H30A	0.9083	0.7598	0.3501	0.036*	
H30B	0.9830	0.8193	0.4154	0.036*	
H30C	0.8611	0.8538	0.3861	0.036*	

### Atomic displacement parameters (Å^2^)

**Table:**

	*U*^11^	*U*^22^	*U*^33^	*U*^12^	*U*^13^	*U*^23^
O1	0.0368 (9)	0.0232 (9)	0.0339 (10)	−0.0136 (8)	−0.0019 (8)	0.0039 (7)
O2	0.0229 (8)	0.0134 (7)	0.0215 (8)	0.0024 (6)	−0.0014 (6)	−0.0003 (6)
O3	0.0264 (8)	0.0221 (8)	0.0205 (8)	0.0031 (7)	0.0032 (7)	−0.0024 (7)
C1	0.0152 (9)	0.0139 (10)	0.0125 (9)	−0.0004 (8)	0.0005 (8)	−0.0016 (8)
C2	0.0207 (9)	0.0159 (10)	0.0148 (10)	−0.0006 (8)	−0.0008 (8)	−0.0018 (8)
C3	0.0266 (11)	0.0153 (10)	0.0195 (10)	−0.0006 (9)	−0.0009 (9)	−0.0049 (8)
C4	0.0173 (9)	0.0163 (10)	0.0234 (11)	0.0010 (8)	−0.0042 (9)	0.0012 (9)
C5	0.0233 (11)	0.0160 (10)	0.0171 (10)	0.0011 (8)	0.0001 (9)	0.0032 (8)
C6	0.0175 (9)	0.0147 (9)	0.0142 (9)	0.0007 (8)	−0.0008 (8)	0.0004 (8)
C7	0.0302 (11)	0.0168 (10)	0.0232 (11)	0.0001 (10)	−0.0095 (10)	0.0006 (9)
C8	0.0416 (14)	0.0241 (12)	0.0230 (12)	−0.0026 (11)	0.0101 (11)	0.0047 (10)
C9	0.0168 (9)	0.0185 (10)	0.0203 (10)	0.0013 (8)	−0.0009 (8)	−0.0004 (9)
C10	0.0282 (11)	0.0168 (10)	0.0146 (10)	−0.0029 (9)	−0.0019 (9)	−0.0017 (8)
C11	0.0296 (11)	0.0157 (10)	0.0160 (10)	0.0018 (9)	0.0005 (9)	−0.0032 (8)
C12	0.0198 (10)	0.0129 (10)	0.0159 (10)	0.0022 (8)	−0.0011 (8)	−0.0004 (8)
C13	0.0138 (9)	0.0137 (10)	0.0124 (9)	−0.0008 (8)	−0.0008 (7)	−0.0006 (8)
C14	0.0209 (10)	0.0169 (10)	0.0257 (11)	−0.0029 (8)	−0.0066 (9)	−0.0010 (9)
C15	0.0152 (9)	0.0181 (10)	0.0146 (10)	−0.0016 (8)	0.0006 (8)	0.0010 (8)
C16	0.0172 (9)	0.0155 (10)	0.0149 (9)	−0.0016 (8)	−0.0004 (8)	0.0004 (8)
C17	0.0159 (9)	0.0158 (10)	0.0139 (9)	−0.0008 (8)	−0.0003 (8)	0.0015 (8)
C18	0.0189 (9)	0.0133 (10)	0.0182 (10)	−0.0013 (8)	0.0041 (8)	−0.0009 (8)
C19	0.0211 (10)	0.0134 (10)	0.0208 (11)	0.0027 (8)	0.0032 (8)	−0.0002 (8)
C20	0.0224 (10)	0.0163 (10)	0.0146 (10)	0.0029 (8)	−0.0013 (8)	−0.0003 (8)
C21	0.0151 (9)	0.0136 (10)	0.0128 (9)	−0.0013 (8)	0.0005 (8)	0.0002 (8)
C22	0.0135 (9)	0.0124 (9)	0.0140 (9)	−0.0002 (7)	−0.0005 (8)	0.0003 (8)
C23	0.0336 (12)	0.0156 (11)	0.0220 (11)	−0.0013 (9)	−0.0010 (10)	−0.0029 (9)
C24	0.0182 (9)	0.0184 (10)	0.0129 (9)	−0.0033 (8)	0.0015 (8)	0.0001 (8)
C25	0.0198 (10)	0.0181 (10)	0.0134 (9)	0.0022 (8)	−0.0005 (8)	0.0009 (8)
C26	0.0264 (11)	0.0133 (10)	0.0187 (11)	0.0031 (9)	0.0011 (9)	0.0011 (8)
C27	0.0182 (9)	0.0150 (10)	0.0166 (10)	−0.0030 (8)	0.0001 (8)	−0.0031 (8)
C28	0.0198 (10)	0.0161 (10)	0.0130 (10)	−0.0006 (8)	0.0009 (8)	−0.0001 (8)
C29	0.0249 (11)	0.0232 (11)	0.0195 (11)	−0.0003 (9)	−0.0051 (9)	−0.0027 (9)
C30	0.0366 (12)	0.0191 (11)	0.0164 (10)	−0.0002 (10)	0.0065 (9)	0.0010 (9)

### Geometric parameters (Å, °)

**Table:**

O1—C4	1.212 (3)	C15—C16	1.533 (3)
O2—C12	1.444 (2)	C15—H15A	0.99
O2—H2	0.841 (10)	C15—H15B	0.99
O3—C27	1.220 (2)	C16—C17	1.557 (3)
C1—C2	1.538 (3)	C16—H16A	0.99
C1—C9	1.548 (3)	C16—H16B	0.99
C1—C6	1.560 (3)	C17—C18	1.527 (3)
C1—C13	1.566 (3)	C17—C22	1.557 (3)
C2—C3	1.535 (3)	C17—H17	1.00
C2—H2A	0.99	C18—C19	1.329 (3)
C2—H2B	0.99	C18—C23	1.507 (3)
C3—C4	1.507 (3)	C19—C20	1.500 (3)
C3—H3A	0.99	C19—H19	0.95
C3—H3B	0.99	C20—C21	1.530 (3)
C4—C5	1.528 (3)	C20—H20A	0.99
C5—C8	1.540 (3)	C20—H20B	0.99
C5—C7	1.545 (3)	C21—C22	1.553 (3)
C5—C6	1.561 (3)	C21—C28	1.567 (3)
C6—C10	1.531 (3)	C21—H21	1.00
C6—H6	1.00	C22—C25	1.535 (3)
C7—H7A	0.98	C22—C24	1.541 (3)
C7—H7B	0.98	C23—H23A	0.98
C7—H7C	0.98	C23—H23B	0.98
C8—H8A	0.98	C23—H23C	0.98
C8—H8B	0.98	C24—H24A	0.98
C8—H8C	0.98	C24—H24B	0.98
C9—H9A	0.98	C24—H24C	0.98
C9—H9B	0.98	C25—C26	1.528 (3)
C9—H9C	0.98	C25—H25A	0.99
C10—C11	1.523 (3)	C25—H25B	0.99
C10—H10A	0.99	C26—C27	1.504 (3)
C10—H10B	0.99	C26—H26A	0.99
C11—C12	1.532 (3)	C26—H26B	0.99
C11—H11A	0.99	C27—C28	1.533 (3)
C11—H11B	0.99	C28—C30	1.535 (3)
C12—C14	1.530 (3)	C28—C29	1.549 (3)
C12—C13	1.550 (3)	C29—H29A	0.98
C13—C15	1.553 (3)	C29—H29B	0.98
C13—H13	1.00	C29—H29C	0.98
C14—H14A	0.98	C30—H30A	0.98
C14—H14B	0.98	C30—H30B	0.98
C14—H14C	0.98	C30—H30C	0.98
			
C12—O2—H2	106 (2)	C16—C15—H15B	109.1
C2—C1—C9	108.35 (16)	C13—C15—H15B	109.1
C2—C1—C6	107.81 (16)	H15A—C15—H15B	107.9
C9—C1—C6	113.99 (17)	C15—C16—C17	114.65 (16)
C2—C1—C13	108.29 (16)	C15—C16—H16A	108.6
C9—C1—C13	111.98 (16)	C17—C16—H16A	108.6
C6—C1—C13	106.21 (15)	C15—C16—H16B	108.6
C3—C2—C1	113.21 (17)	C17—C16—H16B	108.6
C3—C2—H2A	108.9	H16A—C16—H16B	107.6
C1—C2—H2A	108.9	C18—C17—C22	112.49 (16)
C3—C2—H2B	108.9	C18—C17—C16	112.11 (16)
C1—C2—H2B	108.9	C22—C17—C16	112.41 (16)
H2A—C2—H2B	107.8	C18—C17—H17	106.4
C4—C3—C2	112.56 (18)	C22—C17—H17	106.4
C4—C3—H3A	109.1	C16—C17—H17	106.4
C2—C3—H3A	109.1	C19—C18—C23	120.51 (19)
C4—C3—H3B	109.1	C19—C18—C17	121.94 (19)
C2—C3—H3B	109.1	C23—C18—C17	117.44 (18)
H3A—C3—H3B	107.8	C18—C19—C20	124.39 (19)
O1—C4—C3	120.6 (2)	C18—C19—H19	117.8
O1—C4—C5	121.8 (2)	C20—C19—H19	117.8
C3—C4—C5	117.56 (18)	C19—C20—C21	110.95 (17)
C4—C5—C8	108.71 (18)	C19—C20—H20A	109.4
C4—C5—C7	107.11 (17)	C21—C20—H20A	109.4
C8—C5—C7	107.74 (18)	C19—C20—H20B	109.4
C4—C5—C6	109.35 (17)	C21—C20—H20B	109.4
C8—C5—C6	109.75 (18)	H20A—C20—H20B	108.0
C7—C5—C6	114.03 (18)	C20—C21—C22	109.17 (16)
C10—C6—C1	110.45 (16)	C20—C21—C28	113.08 (16)
C10—C6—C5	113.44 (17)	C22—C21—C28	118.11 (16)
C1—C6—C5	117.93 (17)	C20—C21—H21	105.1
C10—C6—H6	104.5	C22—C21—H21	105.1
C1—C6—H6	104.5	C28—C21—H21	105.1
C5—C6—H6	104.5	C25—C22—C24	110.21 (16)
C5—C7—H7A	109.5	C25—C22—C21	108.94 (16)
C5—C7—H7B	109.5	C24—C22—C21	112.03 (16)
H7A—C7—H7B	109.5	C25—C22—C17	107.92 (16)
C5—C7—H7C	109.5	C24—C22—C17	110.47 (16)
H7A—C7—H7C	109.5	C21—C22—C17	107.13 (15)
H7B—C7—H7C	109.5	C18—C23—H23A	109.5
C5—C8—H8A	109.5	C18—C23—H23B	109.5
C5—C8—H8B	109.5	H23A—C23—H23B	109.5
H8A—C8—H8B	109.5	C18—C23—H23C	109.5
C5—C8—H8C	109.5	H23A—C23—H23C	109.5
H8A—C8—H8C	109.5	H23B—C23—H23C	109.5
H8B—C8—H8C	109.5	C22—C24—H24A	109.5
C1—C9—H9A	109.5	C22—C24—H24B	109.5
C1—C9—H9B	109.5	H24A—C24—H24B	109.5
H9A—C9—H9B	109.5	C22—C24—H24C	109.5
C1—C9—H9C	109.5	H24A—C24—H24C	109.5
H9A—C9—H9C	109.5	H24B—C24—H24C	109.5
H9B—C9—H9C	109.5	C26—C25—C22	112.89 (16)
C11—C10—C6	110.33 (17)	C26—C25—H25A	109.0
C11—C10—H10A	109.6	C22—C25—H25A	109.0
C6—C10—H10A	109.6	C26—C25—H25B	109.0
C11—C10—H10B	109.6	C22—C25—H25B	109.0
C6—C10—H10B	109.6	H25A—C25—H25B	107.8
H10A—C10—H10B	108.1	C27—C26—C25	110.62 (16)
C10—C11—C12	113.46 (17)	C27—C26—H26A	109.5
C10—C11—H11A	108.9	C25—C26—H26A	109.5
C12—C11—H11A	108.9	C27—C26—H26B	109.5
C10—C11—H11B	108.9	C25—C26—H26B	109.5
C12—C11—H11B	108.9	H26A—C26—H26B	108.1
H11A—C11—H11B	107.7	O3—C27—C26	121.62 (19)
O2—C12—C14	108.86 (16)	O3—C27—C28	121.16 (18)
O2—C12—C11	108.74 (17)	C26—C27—C28	117.20 (17)
C14—C12—C11	110.49 (18)	C27—C28—C30	109.20 (17)
O2—C12—C13	103.60 (15)	C27—C28—C29	105.32 (17)
C14—C12—C13	115.21 (17)	C30—C28—C29	107.38 (17)
C11—C12—C13	109.57 (16)	C27—C28—C21	111.30 (16)
C12—C13—C15	112.00 (16)	C30—C28—C21	108.59 (16)
C12—C13—C1	115.34 (16)	C29—C28—C21	114.86 (17)
C15—C13—C1	115.11 (16)	C28—C29—H29A	109.5
C12—C13—H13	104.2	C28—C29—H29B	109.5
C15—C13—H13	104.2	H29A—C29—H29B	109.5
C1—C13—H13	104.2	C28—C29—H29C	109.5
C12—C14—H14A	109.5	H29A—C29—H29C	109.5
C12—C14—H14B	109.5	H29B—C29—H29C	109.5
H14A—C14—H14B	109.5	C28—C30—H30A	109.5
C12—C14—H14C	109.5	C28—C30—H30B	109.5
H14A—C14—H14C	109.5	H30A—C30—H30B	109.5
H14B—C14—H14C	109.5	C28—C30—H30C	109.5
C16—C15—C13	112.40 (16)	H30A—C30—H30C	109.5
C16—C15—H15A	109.1	H30B—C30—H30C	109.5
C13—C15—H15A	109.1		
			
C9—C1—C2—C3	69.8 (2)	C1—C13—C15—C16	99.7 (2)
C6—C1—C2—C3	−54.1 (2)	C13—C15—C16—C17	176.18 (17)
C13—C1—C2—C3	−168.58 (17)	C15—C16—C17—C18	−101.6 (2)
C1—C2—C3—C4	54.2 (2)	C15—C16—C17—C22	130.50 (18)
C2—C3—C4—O1	133.3 (2)	C22—C17—C18—C19	−12.3 (3)
C2—C3—C4—C5	−48.9 (3)	C16—C17—C18—C19	−140.2 (2)
O1—C4—C5—C8	−19.3 (3)	C22—C17—C18—C23	171.38 (17)
C3—C4—C5—C8	162.88 (19)	C16—C17—C18—C23	43.5 (2)
O1—C4—C5—C7	96.9 (2)	C23—C18—C19—C20	171.8 (2)
C3—C4—C5—C7	−81.0 (2)	C17—C18—C19—C20	−4.3 (3)
O1—C4—C5—C6	−139.1 (2)	C18—C19—C20—C21	−15.5 (3)
C3—C4—C5—C6	43.1 (2)	C19—C20—C21—C22	50.8 (2)
C2—C1—C6—C10	−175.45 (16)	C19—C20—C21—C28	−175.54 (17)
C9—C1—C6—C10	64.2 (2)	C20—C21—C22—C25	176.90 (16)
C13—C1—C6—C10	−59.6 (2)	C28—C21—C22—C25	45.9 (2)
C2—C1—C6—C5	51.8 (2)	C20—C21—C22—C24	54.7 (2)
C9—C1—C6—C5	−68.5 (2)	C28—C21—C22—C24	−76.3 (2)
C13—C1—C6—C5	167.74 (17)	C20—C21—C22—C17	−66.60 (19)
C4—C5—C6—C10	−177.08 (17)	C28—C21—C22—C17	162.38 (16)
C8—C5—C6—C10	63.8 (2)	C18—C17—C22—C25	163.45 (16)
C7—C5—C6—C10	−57.2 (2)	C16—C17—C22—C25	−68.9 (2)
C4—C5—C6—C1	−45.7 (2)	C18—C17—C22—C24	−76.0 (2)
C8—C5—C6—C1	−164.88 (18)	C16—C17—C22—C24	51.7 (2)
C7—C5—C6—C1	74.2 (2)	C18—C17—C22—C21	46.3 (2)
C1—C6—C10—C11	62.1 (2)	C16—C17—C22—C21	173.97 (15)
C5—C6—C10—C11	−162.95 (18)	C24—C22—C25—C26	67.7 (2)
C6—C10—C11—C12	−57.2 (2)	C21—C22—C25—C26	−55.6 (2)
C10—C11—C12—O2	163.03 (17)	C17—C22—C25—C26	−171.57 (16)
C10—C11—C12—C14	−77.5 (2)	C22—C25—C26—C27	59.3 (2)
C10—C11—C12—C13	50.4 (2)	C25—C26—C27—O3	129.9 (2)
O2—C12—C13—C15	58.4 (2)	C25—C26—C27—C28	−51.7 (2)
C14—C12—C13—C15	−60.4 (2)	O3—C27—C28—C30	−21.1 (3)
C11—C12—C13—C15	174.34 (16)	C26—C27—C28—C30	160.48 (18)
O2—C12—C13—C1	−167.31 (16)	O3—C27—C28—C29	94.0 (2)
C14—C12—C13—C1	73.9 (2)	C26—C27—C28—C29	−84.5 (2)
C11—C12—C13—C1	−51.4 (2)	O3—C27—C28—C21	−140.96 (19)
C2—C1—C13—C12	171.36 (16)	C26—C27—C28—C21	40.6 (2)
C9—C1—C13—C12	−69.2 (2)	C20—C21—C28—C27	−167.41 (17)
C6—C1—C13—C12	55.8 (2)	C22—C21—C28—C27	−38.2 (2)
C2—C1—C13—C15	−55.8 (2)	C20—C21—C28—C30	72.3 (2)
C9—C1—C13—C15	63.6 (2)	C22—C21—C28—C30	−158.42 (17)
C6—C1—C13—C15	−171.39 (16)	C20—C21—C28—C29	−47.9 (2)
C12—C13—C15—C16	−125.90 (18)	C22—C21—C28—C29	81.4 (2)

### Hydrogen-bond geometry (Å, °)

**Table:**

*D*—H···*A*	*D*—H	H···*A*	*D*···*A*	*D*—H···*A*
O2—H2···O3^i^	0.84 (1)	2.15 (1)	2.974 (2)	167 (3)

Symmetry codes: (i) −*x*+2, *y*+1/2, −*z*+3/2.
